# Natural Materials' Potential as Alternative Teeth Remineralization Agents: A Scoping Review

**DOI:** 10.1055/s-0043-1776122

**Published:** 2023-12-04

**Authors:** Irmaleny Irmaleny, Fajar Fatriadi, Christovher Christovher

**Affiliations:** 1 Department of Conservative Dentistry, Faculty of Dentistry, Universitas Padjadjaran, Bandung, Indonesia; 2 Undergraduate Program of Faculty of Dentistry, Universitas Padjadjaran, Bandung, Indonesia

**Keywords:** natural materials, remineralization, teeth, caries

## Abstract

Dental caries is one of the world's major oral health issues. According to The Burden of Disease Study (2016), almost half of the world's population suffers oral health issues, particularly dental caries (3.58 billion individuals). Dental caries treatment through teeth remineralization can be conducted naturally or using remineralization agents. The aim of this study was to map the scientific evidence of natural materials' potential as teeth remineralization agents. The method utilized in this study was the scoping review following the guideline of Preferred Reporting Items for Systematic Reviews and Meta-Analyses Extension for Scoping Review, that is, article searching using search strategies, article screening, data extraction, and conclusion constructing. The searching process was through PubMed, EBSCOhost, and ScienceDirect using keywords and following the inclusion and exclusion criteria. Twenty articles were found and further analyzed. All articles provided the natural materials' potencies in the perspective of antibacterial, increasing saliva pH, remineralization ability, and increasing the hardness of teeth surface. It can be concluded that natural materials can potentially be alternative teeth remineralization agents.

## Introduction

The Global Burden of Disease Study in 2016 stated that oral diseases, dental caries in particular, are suffered by almost half of the people worldwide (±3.58 billion people). The result of Basic Health Research (Riset Kesehatan Dasar/Riskesdas) in 2018 also reported that dental caries/pain/defects were the most common oral disease in Indonesia (45.3%). 1 Dental caries is a multifactorial chronic disease related to the instability of biofilm due to the fermentation of carbohydrates (glucose, fructose, sucrose, and maltose) by specific microorganisms ( *Streptococcus mutans* and *Lactobacillus* ) and the dynamical condition of the demineralization and the remineralization of the teeth's hard tissue. 2 3

Caries is a disease associated with an acid-producing biofilm that starts the teeth demineralization process. 4 The teeth demineralization process is a prolonged instability between pathological and protective factors, thereby leading to the dissolution of apatite crystals in teeth and the deprivation of calcium, phosphate, and other ions. The body's natural reaction toward dental caries is teeth remineralization, which will restore the calcium and phosphate ions supplied from sources outside the body to induce ion deposition to the crystallites on demineralized email. 5

To date, teeth remineralization agents are readily available in the market, for example, casein phosphopeptide-amorphous calcium phosphate (CPP-ACP), fluor, and nano-hydroxyapatite. However, these materials have shortcomings regarding their expensive cost for the lower-middle class economy. 6 Nano-hydroxyapatite as a teeth remineralization agent requires further study to verify its remineralization properties. 7 Other alternative remineralization agents that have been researched as the substitute for chemical materials are natural materials, for example, *Aloe vera* , *Galla chinensis* , siwak ( *Salvadora persica* ), chocolate ( *Theobroma cacao* ), bilimbi ( *Averrhoa bilimbi* ), blood clam shells ( *Anadara granosa* ), soybean ( *Glycine max Merrill* ), rice ( *Oryza sativa* ), and others. Natural materials such as teeth remineralization agents are assumed to have similar potential vis-à-vis the existing teeth remineralization with a more affordable cost. 6 The selection of natural materials in this study was based on the number of articles found and suitable to the determined criteria. Based on the explanation above, it is essential to conduct a study about the potential of natural materials as alternative teeth remineralization agents.

## Method

This present study was a scoping review, a knowledge synthetization following a systematical approach in the evidence-mapping of a topic and identifying the primary concept, sources, and theories. 8 The study began with searching articles on the databases, determining the inclusion and exclusion criteria according to population, concept, and context, performing the Preferred Reporting Items for Systematic Reviews and Meta-Analyses Extension for Scoping Review (PRISMA-ScR) procedures, and extracting the data. 9 The article searching was conducted on three databases, that is, PubMed, EBSCOhost, and ScienceDirect, as seen in Table 1 .

**Table 1:** Searching process in databases using keywords

Search engine	Search strategies	The number of articles
PubMed	aloe vera AND dentistry, averrhoa bilimbi AND dentistry, galla chinensis AND dentistry, theobroma cacao AND dentistry, salvadora persica AND dentistry, Filter: the publication year 2013–2023	263
EBSCOhost	aloe vera AND dentistry, averrhoa bilimbi AND dentistry, galla chinensis AND dentistry, theobroma cacao AND dentistry, salvadora persica AND dentistry, Filter: the publication year 2013–2023	130
ScienceDirect	aloe vera AND dentistry, averrhoa bilimbi AND dentistry, galla chinensis AND dentistry, theobroma cacao AND dentistry, salvadora persica AND dentistry, Filter: the publication year 2013–2023	176

The procedure used the search strategies using AND as the Boolean operators and keywords. The keywords used were: aloe vera AND dentistry, averrhoa bilimbi AND dentistry, galla chinensis AND dentistry, theobroma cacao AND dentistry, salvadora persica AND dentistry. The searching used the filter feature for the year publication of 2013–2023. The article determination was based on the inclusion and exclusion criteria provided in Table 2 .

**Table 2:** Inclusion and exclusion criteria

Inclusion criteria	Exclusion criteria
1. Articles reporting all types of study about the natural materials' potential as teeth remineralization agents	1. Literature review articles
2. Articles published in the last 10 years	2. Article duplicates on different search engine databases
3. Accessible full-text articles	
4. Articles in English or Indonesian and available in the search engine databases	

**Fig. 1:**
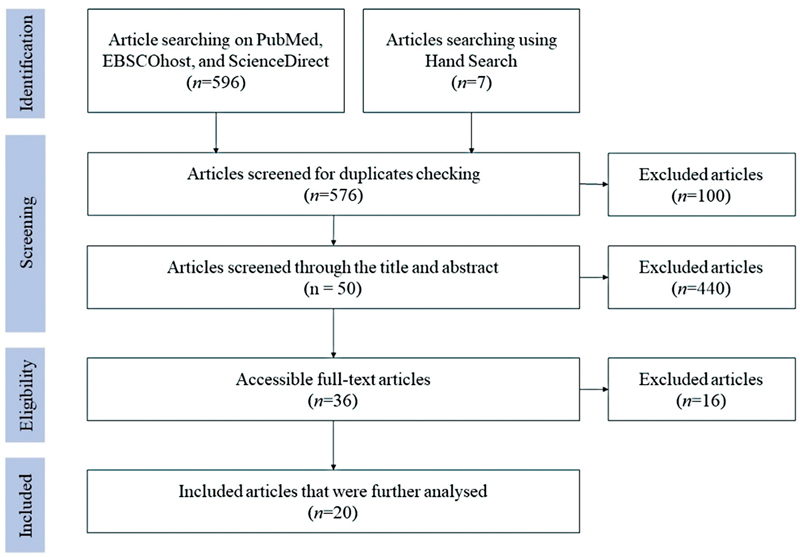
Preferred Reporting Items for Systematic Reviews and Meta-Analyses Extension for Scoping Review (PRISMA-ScR) flowchart.

The article selection was conducted through the screening, eligibility, and stages according to the PRISMA-ScR flowchart, represented in Fig. 1 .

The article selection began with duplicate checking, title or abstract screening, and full-text article screening. The number of articles found from three databases was 569 (263 articles from PubMed, 130 articles from EBSCOhost, and 176 articles from ScienceDirect), and added with 7 articles that were found from hand searching. The total number of articles screened for duplicate checking was 576. After duplicate checking, 100 articles were excluded. The number of articles screened for title or abstract screening was 476. After title or abstract screening, 440 articles were excluded. The 36 remaining articles were read in full text to determine the articles for further analysis. Sixteen articles were excluded, and 20 articles were selected for further analysis.

Further into the analysis, this study demonstrates the potentials that natural materials, for example, *Aloe vera* , *Averrhoa bilimbi* , *Galla chinensis* , *Salvadora persica* , and *Theobroma cacao* , can provide the environment which supports remineralization process to occur. Conditions that were being tested included antimicrobial activity, remineralization ability, increasing saliva pH, and increasing the hardness of teeth surfaces.

## Results

The article screening procedure resulted in 20 included articles for further analysis. The articles were analyzed and transcribed by authors' names, country, publication year, study design, study objectives, type of natural materials, and conclusion ( Table 3 ). The included articles consist of five articles about *Aloe vera* , three about *Averrhoa bilimbi* , four about *Galla chinensis* , four about *Salvadora persica* , and four about *Theobroma cacao* . The natural materials were tested using different study designs, dosage forms, and concentrations to discover their potential in aiding teeth remineralization. The discussion in the table involves the capabilities of each natural material in the teeth remineralization process, that is, antibacterial properties, remineralization ability, increasing saliva pH, and increasing the hardness of teeth surfaces.

**Table 3:** Potentials of natural materials as teeth remineralization agents

Author, country (publication year)	Title	Study design	Study objectives	Natural materials	Conclusion
Pribadi et al, 27 Indonesia (2019)	The Difference in Enamel Surface Hardness after Immersion Process with Cocoa Rind Extract (Theobroma cacao) and Fluoride	Laboratory-based experiment	To analyze the differences in the hardness of the enamel layer hardness between soaking in cacao extract and fluoride	*Theobroma cacao*	The hardness of the enamel layer after soaking in *Theobroma cacao* extract was higher than in fluoride
Balhaddad et al, 23 United States (2021)	Antibacterial Activities of Methanol and Aqueous Extracts of Salvadora persica against Streptococcus mutans Biofilms: An *In vitro* Study	*In vivo*	To analyze the antibacterial property of methanol and aquades extract of *Salvadora persica* against *Streptococcus mutans* biofilm	*Salvadora Persica*	Methanol extract of *Salvadora persica* in the concentration of 10 mg/mL exhibited the ability to inhibit the thickening of the *Streptococcus mutans* biofilm layer
Abidin and Nainggolan, 15 Indonesia (2017)	The Effect of Bilimbi (Belimbing Wuluh) Extract (Averrhoa bilimbi L) Against Dental Remineralisation And Enamel Microstructure ( *In vitro* Research)	*In vitro*	To analyze the enamel remineralization effects of bilimbi extract using SEM and EDX (identifying the enamel microstructure)	*Averrhoa bilimbi*	The *Averrhoa bilimbi L* extract gel, CPP-ACP gel, and a combination of both provided significant effects in enamel remineralization
Sabbagh et al, 25 Saudi Arabia (2020)	The effect of brushing with Salvadora persica (miswak) sticks on salivary Streptococcus mutans and plaque levels in children: a clinical trial	Randomized double-blinded clinical trial	To evaluate the tooth brushing using miswak stick ( *Salvadora persica* ) against *Streptococcus mutans* vis-à-vis the tooth brushing using fluoride toothpaste	*Salvadora persica*	Tooth brushing using *Salvadora persica* stick and fluoride toothpaste significantly reduce plaque scores in students. *Salvadora persica* altered the bacterial proportion in saliva; low-risk of dental caries
Irmaleny et al, 26 Indonesia (2017)	The remineralization potential of cocoa bean extract (Theobroma cacao) to increase the enamel microhardness	*In vitro* experimental	To identify the hardness of the enamel microstructure following remineralization using cacao extract vis-à-vis fluoride	*Theobroma cacao*	A similar hardness of the enamel was obtained in both materials. Both can be used as remineralization agents
Fadel et al, 17 Indonesia (2021)	Uji Aktivitas Antibakteri Obat Kumur Ekstrak Daun Belimbing Wuluh (Averrhoa Bilimbi L.) Terhadap Bakteri Streptococcus mutans Penyebab Karies Gigi	Experimental	To analyze the practicality of bilimbi leaves ( *Averrhoa bilimbi L.* ) extract as a mouthwash against *Streptococcus mutans*	*Averrhoa bilimbi*	The mouthwash formulation of the *Averrhoa bilimbi L* extract suppressed the growth of *Streptococcus mutans*
Yuanita et al, 6 Indonesia (2020)	Enamel hardness differences after topical application of theobromine gel and Casein Phosphopeptide-amorphous Calcium Phosphate	*In vitro* laboratory experimental	To compare the hardness of enamel between the application of CPP-ACP and theobromine gel	*Theobroma cacao*	The hardness of enamel after the application of theobromine gel was higher vis-à-vis CPP-ACP
Abhary and Al-Hazmi, 22 Saudi Arabia (2016)	Antibacterial activity of Miswak (Salvadora persica L.) extracts on oral hygiene	Experimental	To discover the antimicrobial agents in the *Salvadora persica* extract	*Salvadora persica*	*Salvadora persica* possessed broad antibacterial effects against Gram-positive and negative and had > 1 antimicrobial agents
Yikici and Ozcan, 14 Turkey (2022)	Remineralization Activities of Toothpastes with and without Aloe Vera with Different Ratios of Fluoride on Demineralized Enamel: An In-vitro Study	*In vitro*	To evaluate and compare the efficiency of *Aloe vera* and commercial toothpaste with different levels and types of fluoride on initial enamel lesion (artificial)	*Aloe vera*	Fluoride toothpaste was effective against teeth demineralization. *Aloe vera* increased the remineralization efficiency of high-fluoride toothpaste
Hajiahmadi et al, 10 Iran (2021)	Comparative Evaluation of Antibacterial Effect of Propolis and Aloe Vera, Xylitol, and Cpp-Acp Gels on Streptococcus mutans and Lactobacillus *in vitro*	*In vitro* experimental	To identify the antibacterial property of a gel comprised of propolis and *Aloe vera* , fluoride, xylitol, and CPP-ACP	*Aloe vera*	Propolis/ *Aloe vera* /fluoride/xylitol gel inhibited the growth of *Streptococcus mutans* and *Lactobacillus* . The antibacterial property of propolis */Aloe vera* gel was better vis-à-vis other gels; the property was still maintained in a low concentration
Al Haddad et al, 12 Lebanon (2021)	Comparison of the Remineralizing Effect of Brushing with Aloe vera versus Fluoride Toothpaste	*In vitro*	To analyze the comparison of remineralization effects after tooth brushing using 3 toothpaste and *Aloe vera* gel	*Aloe vera*	The remineralization effect of fluoride toothpaste, *Aloe vera* gel, and *Aloe vera* paste were similar
Abdel-Azem et al, 18 Egypt (2020)	Effect of Galla Chinensis on Remineralization of Early Dentin Lesion	*In vitro*	To analyze the effectiveness of *Galla chinensis* extract in dentin remineralization	*Galla chinensis*	*Galla chinensis* extract could be used as an alternative dentin remineralization agent
Tang et al, 21 China (2015)	Effects of gallic acid on the morphology and growth of hydroxyapatite crystals	Experimental	To discover the effect of gallic acid (one of the chemical components of *Galla chinensis* ) on the morphology and formation of hydroxyapatite crystals	*Galla chinensis*	Gallic acid was attributed to the crystal formation due to the interaction between gallic acid and Ca ^2+^
Zhang et al, 19 China (2016)	Galla chinensis Compounds Remineralize Enamel Caries Lesions in a Rat Model	*In vitro*	To discover the effects of chemical components in *Galla chinensis* toward enamel caries remineralization in rats	*Galla chinensis*	*Galla chinensis* remineralized the enamel caries in rats
Jain et al, 13 India (2016)	Antibacterial Effect of Aloe Vera Gel against Oral Pathogens: An In-vitro Study	*In vitro*	To discover the antimicrobial and inhibition effects of *Aloe vera* gel in various concentrations against oral pathogen bacteria	*Aloe vera*	*Aloe vera* gel could be used as an alternative antibacterial agent for oral diseases (in a high concentration)
Bhati et al, 11 India (2015)	Evaluation of antimicrobial efficacy of Aloe vera and Meswak containing dentifrices with fluoridated dentifrice: An *in vivo* study	*In vivo*	To compare the antimicrobial effects between fluoride and herbal toothpaste	*Aloe vera* and *Salvadora persica*	Toothpaste containing *Aloe vera* and siwak could be used as an alternative to fluoride toothpaste in terms of the antimicrobial effect
Kim and Jin, 20 Korea (2018)	Galla chinensis extracts and calcium induce remineralization and antibacterial effects of enamel in a Streptococcus mutans biofilm model	*In vitro*	To identify the effects of *Galla chinensis* extract and calcium (Ca) combination in enamel remineralization	*Galla chinensis*	*Galla chinensis* increased enamel remineralization significantly and was more intense after adding Ca. The extract possessed excellent bactericidal properties, lowered acid production, and altered the microstructure of *Streptococcus mutans*
Balto et al, 24 Saudi Arabia (2017)	Effectiveness of Salvadora persica extracts against common oral pathogens	Laboratory experimental	To evaluate the antibacterial activity of ethanol and hexane extracts of *Salvadora persica* against common oral pathogens	*Salvadora persica*	High concentration of ethanol and hexane extract of *Salvadora persica* yielded antimicrobial effects against *Streptococcus mutans, Streptococcus salivarius,* and *Streptococcus sanguinis*
Restuning et al, 16 Indonesia (2022)	Pengaruh Rebusan Daun Belimbing Wuluh (Averrhoa Bilimbi Linn) Terhadap pH Saliva	Quasi-experimental	To discover the effects of before and after using the decoction of bilimbi leaf gargle	*Averrhoa bilimbi*	Bilimbi leaf decoction water exhibited excellent effects in saliva pH mean score
Fajriani et al, 28 Indonesia (2016)	The role of cacao extract in reduction of the number of mutans streptococci colonies in the saliva of 12–14-year-old-children	Cross-sectional study design with time-series experimental study	To discover the effectiveness of ethanol extract of cacao bean in reducing *Streptococcus* colonies in children's saliva in mouthwash dosage form	*Theobroma cacao*	Gargling using cacao bean extract mouthwash reduced the *Streptococcus mutans* colonies effectively

Abbreviations: CPP-ACP, casein phosphopeptide-amorphous calcium phosphate; EDX, energy-dispersive X-ray spectroscopy; SEM, scanning electron microscopy.

## Discussion

Natural materials are potentially teeth remineralization agents in various perspectives, for example, antimicrobial properties, remineralization ability, increasing saliva pH, and increasing the hardness of teeth surface. All articles included in this study reported natural materials' potencies in supporting teeth remineralization. The analysis results provided information that natural materials ( *Aloe vera* , *Averrhoa bilimbi* , *Galla chinensis* , *Salvadora persica* , and *Theobroma cacao* ) could be used as alternative teeth remineralization agents.

The studies have proven that *Aloe vera* could be utilized as a teeth remineralization agent due to its antibacterial property and remineralization ability. 10 11 12 13 14 Yikici and Ozcan stated that *Aloe vera* intensified the remineralization efficiency of toothpaste in demineralized enamel. Their study show that lesser active components can be found in commercially available toothpaste through processing, therefore making *Aloe vera* gel when applied directly better in aiding remineralization. 14 Al Haddad et al reported that the teeth remineralization outcomes from *Aloe vera* gel, fluoride toothpaste, and *Aloe vera* /fluoride toothpaste did not differ much. Polyphenols in *Aloe vera* gel is likely to be the active components making the gel acts as a dental remineralizing booster. 12 *Aloe vera* was also discovered to possess antibacterial effects against oral pathogen bacteria in a study by Jain et al and subsequently supported by studies by Hajiahmadi et al and Al Haddad et al, which reported that *Aloe vera* inhibited the growth of *Streptococcus mutans* and *Lactobacillus* —oral pathogen bacteria. 10 12 13 Hajiahmadi et al also mentioned that propolis and *Aloe vera* gel yielded better antibacterial effects vis-à-vis CPP-ACP. 10 According to Bhati et al, *Aloe vera* could be an alternative to fluoride toothpaste in terms of antimicrobial properties. 11

In many studies, bilimbi ( *Averrhoa bilimbi* ) was found to generate a remineralization effect, antibacterial effect, and increase saliva pH; therefore, it can be used as a teeth remineralization agent. 15 16 17 According to Abidin and Nainggolan, *Averrhoa bilimbi* extract yielded significant effects in the enamel remineralization and produced an enamel layer with smaller porous in demineralized teeth. The study also reported that the combination of *Averrhoa bilimbi* extract and CPP-ACP induced enamel remineralization closer to normal conditions. Abidin and Nainggolan provided that H ^+^ ion in *Averrhoa bilimbi* extract when applied will bind into HPO _4_^2−^ in saliva and furthermore binds with Ca ^2+^ from the extract will produce CaHPO _4_ as a result that will diffuse into the teeth surface enhancing the remineralization process. 15 Fadel et al stated that *Averrhoa bilimbi* extract could suppress the growth of *Streptococcus mutans* , which is the causative agent of dental caries. 17 Based on a study by Restuning et al, the bilimbi leaves boiled water could also increase saliva pH (6.73). 16

Some studies reported that *Galla chinensis* , a Chinese herb, could be applied as a teeth remineralization agent due to its remineralization ability and antibacterial effect. 18 19 20 21 Zhang et al stated that the chemical components of *Galla chinensis* could induce enamel caries remineralization in rats. Remineralization that *Galla Chinensis* provides are different than fluoride because enamel organic matrix plays a decent role in assisting the process as they will make a large complex becoming calcium ion carrier. 19 Abdel-Azem et al, in their study, added the fact that *Galla chinensis* possessed remineralization ability on dentin lesions. Component contained within *Galla chinensis* extract from the complex “dentin organic matrix – Galla Chinensis Extract – Ca ^2+^ ” can diffuse into dentin lesions providing remineralization as it delivers Ca ^2+^ ions and helps in mineral ions deposition into the surface. Their study also compared *Galla chinensis* and NaF, and it was discovered that *Galla chinensis* possessed remineralization ability after 1 minute of soaking. 18 A study by Kim and Jin reported that *Galla chinensis* significantly increased enamel remineralization, led to bactericidal activity against *Streptococcus mutans* , and could maintain a pH level of 7. 20 Tang et al pointed out that one of the chemical components of *Galla chinensis* was gallic acid, which attributed in the hydroxyapatite-resembling crystal formation after 3 days of application. 21

Siwak ( *Salvadora persica* ) could be used as a teeth remineralization agent due to its remineralization ability and antibacterial effect. 11 22 23 24 A study by Bhati et al stated that *Salvadora persica* can be used as a substitute for fluoride toothpaste due to its antibacterial properties. 11 Balto et al and Abhary and Al-Hazmi also reported that the antimicrobial property of *Salvadora persica* was effective against various oral pathogens and was classified as a broad-spectrum antimicrobial. Additionally, Balto et al stated that active components such as isothiocyanates provide antibacterial property which results in a better environment on the oral space. The study that Abhary and Al-Hazmi conducted using *Salvadora persica* in preparation as a mouth wash supports the antimicrobial activity that contains in it. 22 24 The results from studies by Sabbagh et al and Balhaddad et al also mentioned that *Salvadora persica* possessed antimicrobial properties, particularly against *Streptococcus mutans* . 23 25

Some studies have investigated cacao ( *Theobroma cacao* ) and the results verified that *Theobroma cacao* could be implemented as a teeth remineralization agent due to its remineralization ability, antibacterial effect, and evidence to increase teeth surface. 6 26 27 28 Fajriani et al mentioned that *Theobroma cacao* extract could reduce the amount of *Streptococcus mutans* colonies in children. 28 According to Irmaleny et al, *Theobroma cacao* could increase the hardness of enamel microstructure, supported by a study by Pribadi et al that compared cacao extract with fluoride. Additionally, Irmaleny et al stated that direct application of theobromine inside the cocoa extract will form interstitial reaction to substitute the loss of hydroxyapatite crystal, which in other words can describe the remineralization process that it can provide. 26 27 Yuanita et al, in their study, stated that *Theobroma cacao* gel was better at strengthening the enamel vis-à-vis CPP-ACP. 6 The usage of *Theobroma cacao* resulted in an increase in enamel hardness by up to 40%. 6 Other studies calculated the increase of enamel hardness of CPP-ACP, and it was discovered that the increase ranged around 33%, which amplifies that *Theobroma cacao* extract yields a better outcome. 29 30 31

The limitation of this scoping review involves the heterogeneous articles regarding the samples, the study designs, and the results due to the inclusion and exclusion criteria. Therefore, further study is required to analyze natural materials' type, dosage form, and effectiveness. Nevertheless, the natural materials selected in this study can be the scientific evidence of natural materials being alternatives for teeth remineralization agents.

## Conclusion

Natural materials are potentially utilized as dental mineralization agents due to their properties in presenting an antibacterial effect, increasing saliva pH, and increasing the hardness of enamel layer.
